# Factors affecting in-stent restenosis after angioplasty with the Enterprise stent for intracranial atherosclerotic diseases

**DOI:** 10.1038/s41598-021-89670-x

**Published:** 2021-05-18

**Authors:** Kun Zhang, Tian-Xiao Li, Zi-Liang Wang, Bu-Lang Gao, Jian-Jun Gu, Hui-Li Gao, Yong-Feng Wang, Jin-Chao Xia

**Affiliations:** Henan Provincial Cerebrovascular Hospital, Henan Provincial People’s Hospital, Zhengzhou University, 7 Weiwu Road, Zhengzhou, 450003 Henan Province China

**Keywords:** Cerebrovascular disorders, Stroke

## Abstract

This study investigated factors affecting the safety and in-stent restenosis after intracranial stent angioplasty using the Enterprise stent for symptomatic intracranial atherosclerotic stenosis. Between January 2017 and March 2019, patients with intracranial atherosclerotic stenosis treated with Enterprise stent angioplasty were enrolled, including 400 patients in the modeling group and 89 patients in the validation group. The clinical factors affecting in-stent restenosis after Enterprise stent angioplasty in the modeling group were analyzed, and a logistic regression model of these factors was established and validated in the validation group. The receiver operating characteristic (ROC) curve and the area under the ROC curve (AUC) were analyzed. In the modeling group with 400 patients, there were 410 lesions, including 360 stenotic lesions and 50 occluded lesions, with 176 (42.9%) lesions in the anterior circulation and 234 (57.1%) in the posterior circulation. Successful stenting was performed in 398 patients (99.5%). Stenosis was significantly (*P* < 0.05) improved after stenting compared with before stenting (27.7% ± 2.9% vs. 77.9% ± 8.0%). Periprocedural complications included ischemic stroke (3.25%), hemorrhagic stroke (0.75%), and death (0.50%), with a total periprocedural complication rate of 4.0%. The first follow-up angiography was performed in 348 (87.0%) patients with 359 lesions 3.5–14 months (mean 5.7 months) after stenting. In-stent restenosis occurred in 62 (17.3%) lesions, while the other 295 (82.7%) had no restenosis. Lesion location, calcification degree, balloon expansion pressure, residual stenosis, intraprocedural dissection, and cerebral blood flow TICI grade were significant (*P* < 0.05) risk factors for in-stent restenosis. The in-stent restenosis prediction model was established as follows: *P* = 1/[1 + e^−(−6.070–1.391 location + 2.745 calcification + 4.117 balloon inflation pressure + 2.195 intraprocedural dissection + 1.163 residual stenosis + 1.174 flow TC grade)^]. In the validation group, the AUC in the ROC curve analysis was 0.902 (95% CI: 0.836–0.969), and when the cutoff value was 0.50, the sensitivity and specificity of this model were shown to be 76.92% and 80.26%, respectively, in predicting in-stent restenosis at angiographic follow-up, with a total coincidence rate of 79.78%. In conclusion, in-stent restenosis after intracranial Enterprise stenting is affected by stenosis location, calcification, balloon inflation pressure, intraprocedural arterial dissection, residual stenosis, and cerebral flow grade, and establishment of a logistic model with these factors can effectively predict in-stent restenosis.

## Introduction

Intracranial arterial stenosis (ICAS) is currently a major cause of ischemic stroke and affects almost 30% of Chinese patients who have cerebral ischemia^1,2^; furthermore, patients with symptomatic intracranial atherosclerotic stenosis greater than 70% have a stroke risk of 12.2% per year^3–5^. Stent angioplasty with balloon dilatation followed by stent deployment has been an effective therapeutic option for intracranial atherosclerotic stenosis, and compared with deployment of balloon-expandable coronary stents for this kind of stenosis, the Wingspan stent (Boston Scientific, Fremont, CA, USA) could significantly increase procedural safety and eliminate most of the access and sizing issues^6^. However, a high rate (31%) of in-stent restenosis and perioperative complications has been reported in angiographic and clinical follow-up^6–9^, and the greater radial force of the Wingspan stent is likely to have caused perioperative complications, intimal hyperplasia, and subsequently, in-stent restenosis. Based on this suspicion, researchers began treating intracranial stenoses by moderately undersized balloon dilatation followed by implantation of a slightly oversized self-expandable stent, as suggested by Bose et al^10^, using the Enterprise stent (Codman Neurovascular, Raynham, MA, USA) in particular^4,9,11–14^. The Enterprise stent is a self-expanding closed-cell stent that was originally designed for assisted coiling of wide-necked intracranial aneurysms^15^. This stent appears to perform better than the Wingspan stent for complex intracranial atherosclerotic stenoses due to its high flexibility, decreased radial force, special carrier system structure, and sufficiency to prevent elastic recoil and in-stent restenosis^4,16^. Even though the Enterprise stent has less severe in-stent restenosis after deployment for treating intracranial atherosclerotic lesions, restenosis still occurs within the stent. Currently, however, no studies have been performed to investigate factors affecting restenosis within the Enterprise stent after intracranial deployment for atherosclerotic stenosis. This study was consequently conducted to investigate possible factors affecting restenosis within the Enterprise stent after deployment to treat intracranial atherosclerotic lesions.

## Results

**Figure 1:**
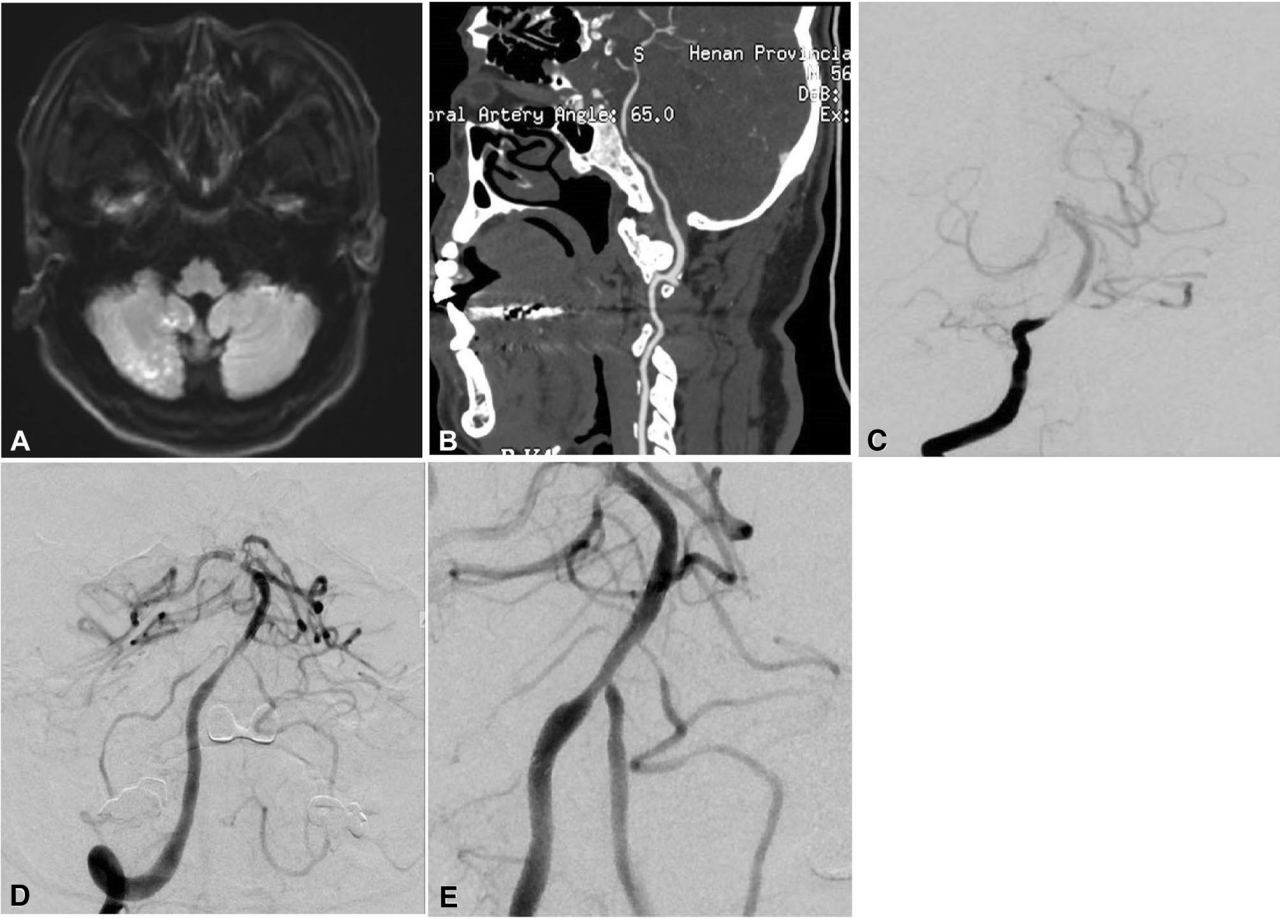
Severe stenosis at the lower segment of the basilar artery. (**A**) Magnetic resonance imaging (DWI) showed disperse lesions of new infarction in the right hemisphere of the cerebellum. (**B**) Computed tomography demonstrated severe stenosis at the lower segment of the basilar artery without calcification. (**C**) Digital subtraction angiography revealed severe stenosis at the lower segment of the basilar artery. (**D**) An Enterprise stent was deployed at the stenosis leading to patent flow. (**E**) Follow-up angiography at 6 months after stenting demonstrated good patency of the basilar artery at the lesion.

**Figure 2:**
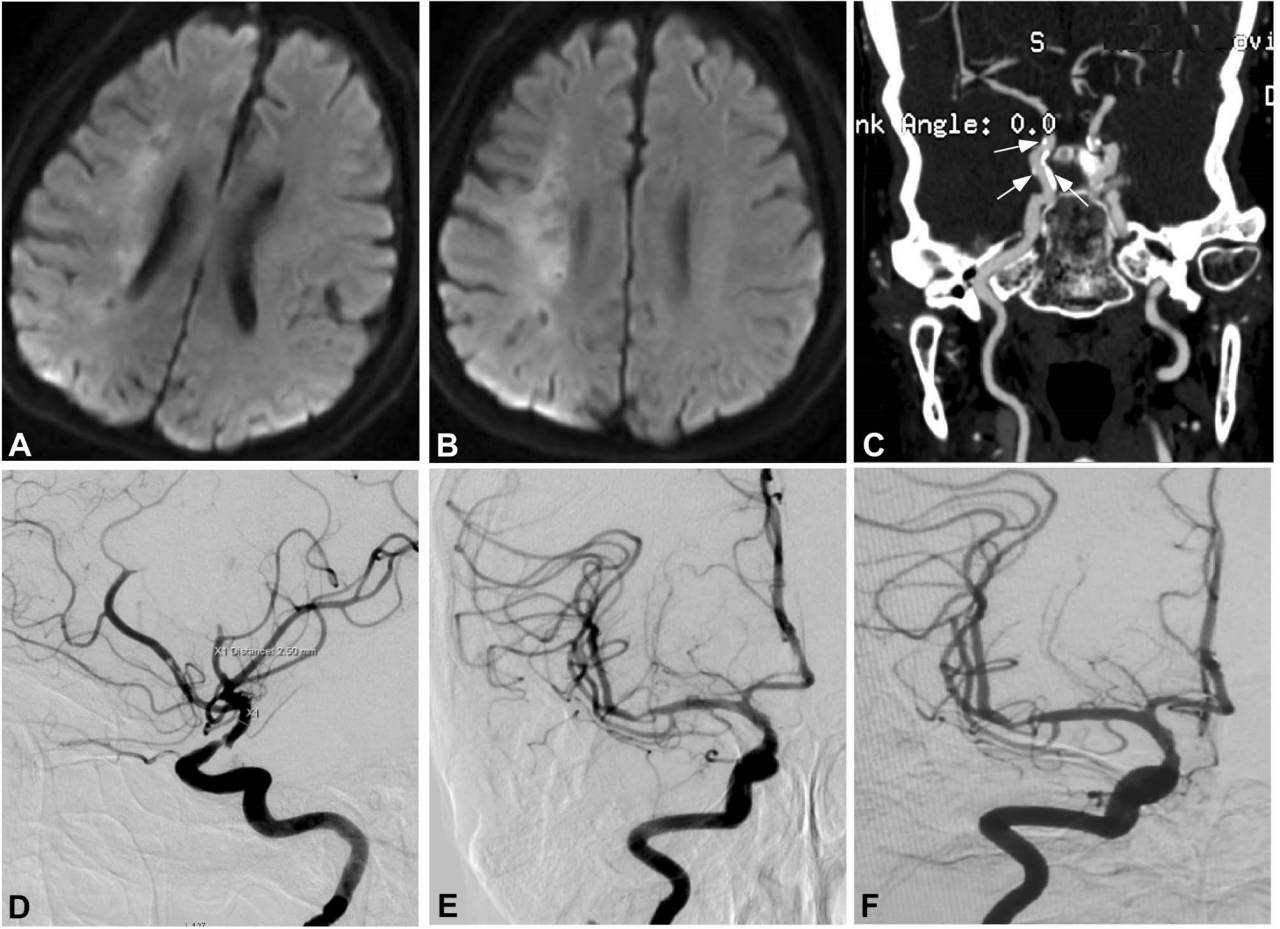
Right internal carotid artery severe stenosis. (**A**) Magnetic resonance imaging (MRI) showed an ischemic infarct at three days after onset. (**B**) MRI at 14 days after onset revealed increased area of the infarction. (**C**) Computed tomography revealed calcification (arrows) around the posterior communicating artery segment of the right internal carotid artery. (**D**) Cerebral digital subtraction angiography demonstrated severe stenosis at the posterior communicating artery segment of the right internal carotid artery. (**E**) Stent angioplasty was performed with an Enterprise stent and the severe stenosis disappeared. (**F**) Nine months after stent angioplasty, the stent remained patent.

**Figure 3:**
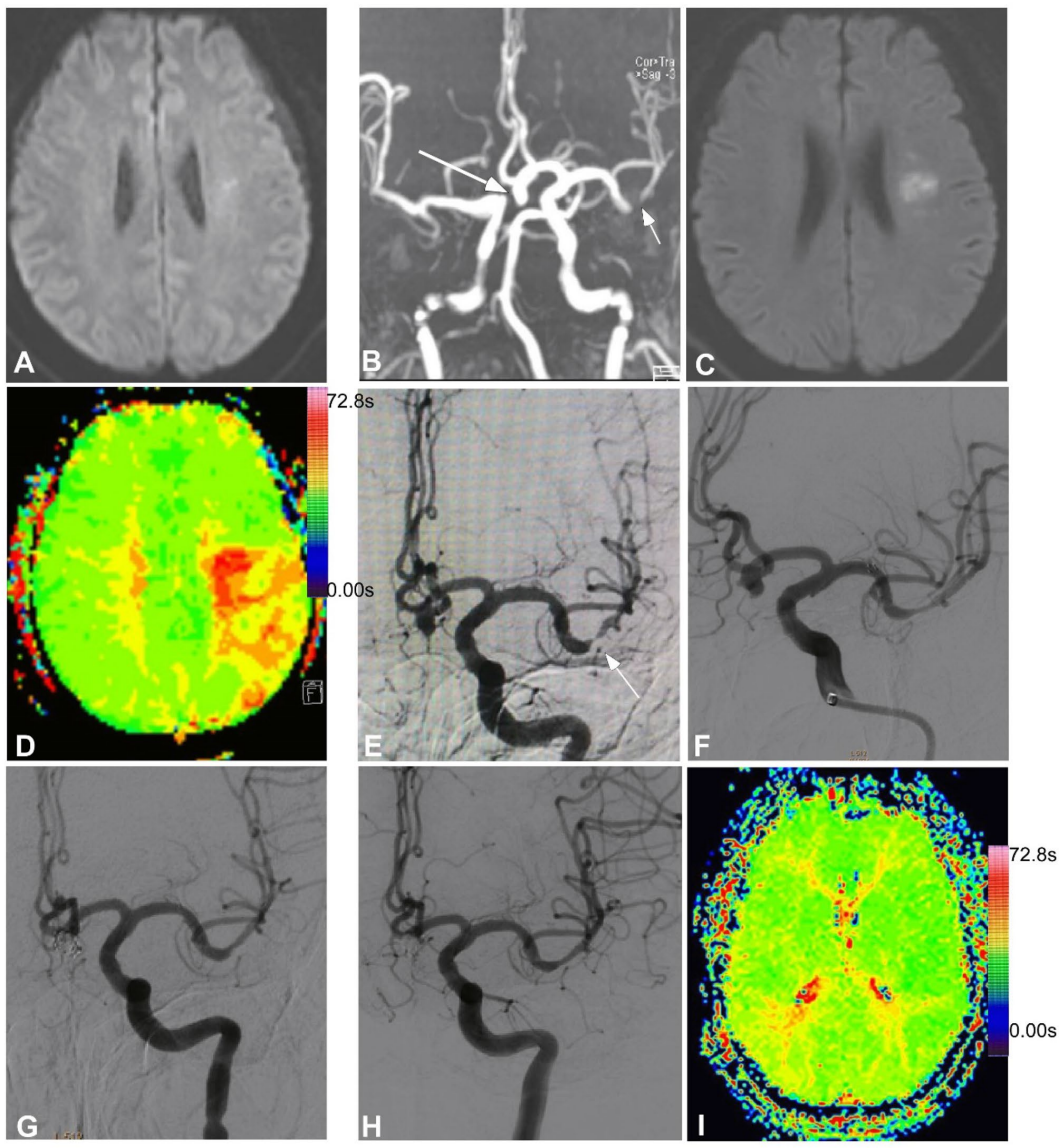
Severe stenosis in the lower trunk of the left middle cerebral artery (MCA). (**A**) Magnetic resonance imaging (MRI) showed bead-like high intensity lesion near the ventricular body. (**B**) MRI angiography revealed severe stenosis in the lower trunk of the left MCA (small arrow) and an aneurysm (large arrow) at the anterior communicating artery. (**C**) Nine days after admission, the symptoms were aggravated and the second MRI scan demonstrated increased infarction area. (**D**) Perfusion imaging showed that greater time is needed for the blood flow to reach the left parietal occipital lobe. E&F. Digital subtraction angiography demonstrated severe stenosis in the lower trunk of the left MCA (arrow, **E**), and stent angioplasty was performed with an Enterprise stent to relieve the stenosis (**F**). **G**. Endovascular coil embolization was performed to completely occlude the anterior communicating aneurysm. (**H**). Follow-up angiography at six months following stenting revealed patent flow in the lower trunk of the left MCA. (**I**). Perfusion imaging showed the time is significantly improved for the blood flow to reach the left parietal occipital lobe.

**Figure 4:**
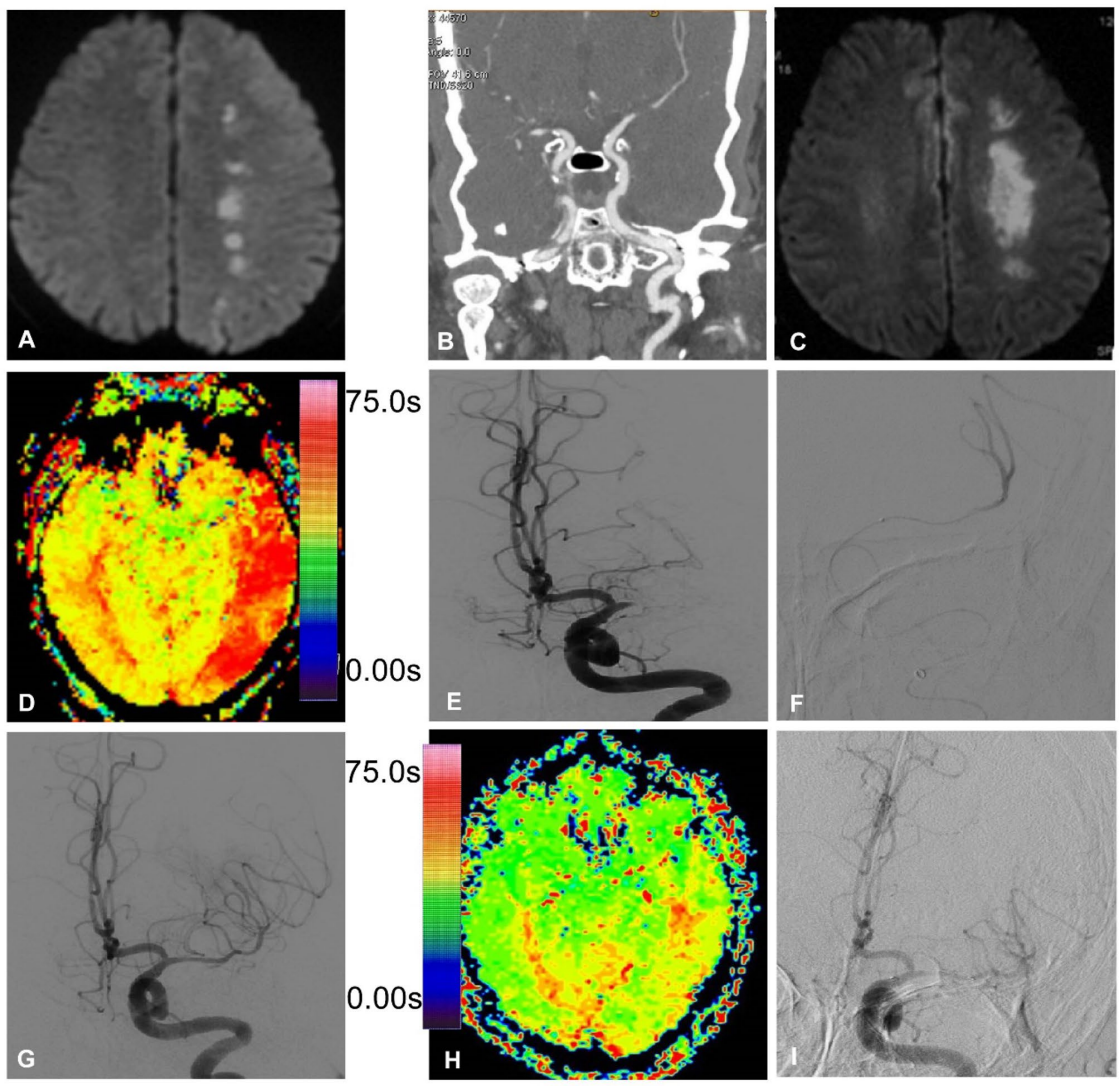
Endovascular recanalization of occluded left middle cerebral artery (MCA) and angiographic follow-up. (**A**) DWI of magnetic resonance imaging (MRI) immediately after admission revealed bead-like high signal in the left hemisphere. (**B**) Computed tomography angiography showed occluded M1 segment of the left MCA. (**C**) Two days after admission, DWI of MRI demonstrated increased area of high signal lesions in the left hemisphere with fusion of the original bead-like lesions. (**D**) PWI of MRI revealed greater time needed for the blood flow to reach the left hemisphere. (**E**) Digital subtraction angiography showed occluded M1 segment of the left MCA with no display of the distal arterial branches. (**F**) A microguidewire was used to explored the occluded M1 segment, and a microcatheter was navigated along the microguidewire through the occluded M1 segment into the M2 segment of the left MCA, and angiography through this microcatheter indicated patency of distal arterial branches. (**G**) An Enterprise 4.5 mm*22 mm stent was deployed at the occluded segment after balloon angioplasty, with recanalized arterial branches of the left MCA. (**H**) PWI of MRI demonstrated improved blood flow to the left hemisphere, much better than before recanalization. (**I**) Digital subtraction angiography six months later revealed patent left MCA with no instent stenosis.

The modeling group comprised 400 patients, including 164 females and 236 males, with a mean age of 59.22 ± 9.11 years. For validation of the model, another 89 patients were enrolled, including 33 females and 56 males with a mean age of 58.42 ± 7.26 years (Table 1). No significant (*P* > 0.05) differences existed in the baseline data between the two groups. In the modeling group, there were 410 lesions, including 360 stenotic lesions and 50 occluded lesions (Table 1). The mean length of the lesions was 13.02 ± 0.78 mm (range 6–30 mm), with 176 (42.9%) lesions in the anterior circulation and 234 (57.1%) in the posterior circulation. Successful stent angioplasty was performed in 398 patients with a success rate of 99.5% and a mean duration for stent angioplasty of 74.32 ± 16.29 min (range 42–175 min) (Figs. 1, 2, 3, and 4). The procedure failed in two patients. In one patient, the balloon catheter could not be navigated through the stenosis, and in the other patient, the balloon could not be fully inflated to expand the stenosis because of severe calcification in the lesion. Stenosis was significantly (*P* < 0.05) improved after stent angioplasty compared with before stent angioplasty (27.7% ± 2.9% vs. 77.9% ± 8.0%). During the 30-day periprocedural period, ischemic stroke occurred in 13 patients (3.25%), hemorrhagic stroke in three patients (0.75%), and death in two patients (0.50%), with a total periprocedural complication rate of 4.0%.

**Table 1:** Clinical data of patients in the modelling and validation groups with intracranial atherosclerotic diseases.

Variables		Modelling	Validation	*P* value
Patients	Sex (F/M)	164/236	33/56	0.495
	Age (y)	59.22 ± 9.11 (34–79)	58.42 ± 7.26 (31–77)	0.775
Co-morbidities	Diabetes mellitus	162(40.5%)	40(44.9%)	0.441
	Hypertension	171(42.8%)	41(46.1%)	0.568
	Hyperlipemia	108 (27.0%)	22 (24.7%)	0.660
	High homocysteine	30(7.5%)	12(13.5%)	0.068
	Coronary heart disease	203(50.8%)	36(40.4%)	0.348
	Past cerebral infarction	166(41.5%)	45(50.6%)	0.119
Lesion types	Stenosis lesions	360 (87.8%)	78(87.6%)	0.669
	Symptomatic stenosis	312 (76.1%)	69 (77.5%)	
	Non-symptomatic stenosis	48 (11.7%)	9 (10.1%)	
	Occlusive disease	50 (12.2%)	11 (12.4%)	0.966
Lesion location	Anterior circulation	176 (44.0%)	40(44.9%)	0.871
	Posterior circulation	224 (56.0%)	49 (55.1%)	
Stenosis degree	Prestenting	77.9% ± 8.0% (65%-90%)	77.2% ± 7.3% (60%-90%)	0.758
	Poststenting	27.7% ± 2.9% (10%-50%)	28.0% ± 4.4% (10%-40%)	-0.794

No significant (*P* > 0.05) difference existed in the baseline data between the two groups.

The first follow-up angiography was performed in 348 (87.0%) patients with 359 lesions, with a follow-up time ranging from 3.5–14 months (mean 5.7 months). In-stent restenosis occurred in 62 (17.3%) lesions, while the other 297 (82.7%) lesions had no restenosis.

Single-factor analysis showed that the location of the lesion, calcification degree (with 1/2 circular calcification as the borderline), balloon expansion pressure, residual stenosis, intraprocedural dissection, and cerebral blood flow TICI grade were significant (*P* < 0.05) risk factors for in-stent restenosis at follow-up after Enterprise angioplasty (Table 2). However, age (with 50 years as the borderline), smoking history, hypertension, alcoholic abuse, diabetes mellitus, dyslipidemia, coronary heart disease, hyperhomocysteinemia and pre-expansion time were not significant (*P* > 0.05) risk factors for in-stent restenosis (Table 2).

**Table 2:** Single factor analysis of risk factors for in-stent restenosis.

Group		Follow-up angiography		*X*^2^	*P* value
		Patent	Restenosis		
Location	BA&MCA	154(91.7%)	14(8.3%)	316.8	< 0.0001
	VA&ICA	144(75.4%)	47(24.6%)		
Calcification	> 1/2 circle	41(52.6%)	37(47.4%)	65.5	< 0.0001
	≤ 1/2 circle	257(91.5%)	24(8.5%)		
Balloon inflation pressure	> 4 ATM	46(47.4%)	51(52.6%)	119.3	< 0.0001
	≤ 4 ATM	252(96.2%)	10(3.8%)		
Intraprocedural dissection	Yes	52(56.5%)	40(43.5%)	61.5	< 0.0001
	No	246(92.1%)	21(7.9%)		
Residual stenosis	> 30%	40(43.0%)	53(57.0%)	142.4	< 0.0001
	≤ 30%	258(97.0%)	8(3.0%)		
TICI	> 2b	51(61.4%)	32(38.6%)	35.6	< 0.0001
	≤ 2b	247(89.5%)	29(10.5%)		
Age	> 50y	173(82.0%)	38(18.0%)	0.376	0.5398
	≤ 50y	125(84.5%)	23(15.5%)		
Smoking history	Yes	140(80.9%)	33(19.1%)	1.028	0.3107
	No	158(84.9%)	28(15.1%)		
Alcoholic abuse	Yes	104(84.6%)	19(15.4%)	0.316	0.5738
	No	194(82.2%)	42(17.8%)		
Hypertension	Yes	249(82.5%)	53(17.5%)	0.420	0.5170
	No	49(86.0%)	8(14.0%)		
Diabetes mellitus	Yes	197(81.7%)	44(18.3%)	0.833	0.3615
	No	101(85.6%)	17(14.4%)		
Dyslipidemia	Yes	266(84.1%)	50(15.8%)	2.556	0.1099
	No	32(74.4%)	11(25.6%)		
Coronary heart disease	Yes	140(80.9%)	33(19.1%)	1.028	0.3107
	No	158(84.9%)	28(15.1%)		
Hyperhomocysteinemia	Yes	104(84.6%)	19(15.4%)	0.316	0.5738
	No	194(82.2%)	42(17.8%)		
Pre-expansion time	> 30 s	173(82.0%)	38(18.0%)	0.376	0.5398
	≤ 30 s	125(84.5%)	23(15.5%)		

BA, basilar artery; MCA, middle cerebral artery; VA, vertebral artery; ICA, internal carotid artery; TICI, thrombolysis in cerebral infarction grade.

Multivariate logistic regression analysis including the above significant risk factors demonstrated that lesion location (vertebral artery V4 segment, basilar artery, middle cerebral artery, and internal carotid artery siphon segment), calcification (with 1/2 circular calcification as the borderline), balloon inflation pressure, intraprocedural arterial dissection, residual stenosis and cerebral flow TICI grade were the dominant risk factors (*P* < 0.05) (Table 3).

**Table 3:** Regression model of instent restenosis based on imaging and clinical features of lesions.

Indexes	Regression coefficient estimated value (B)	*P* value	Dominance ratio	95%CI
Constant	− 6.070	0.000	0.002	
Location	− 1.391	0.005	0.249	0.095–0.652
Calcification	2.745	0.000	15.564	5.61–43.21
Balloon inflation pressure	4.117	0.000	61.367	17.757–212.080
Intraprocedural dissection	2.195	0.000	8.982	3.132–25.763
Residual stenosis	1.163	0.013	3.199	1.284–7.973
TICI	1.174	0.012	3.234	1.291–8.101

**Figure 5:**
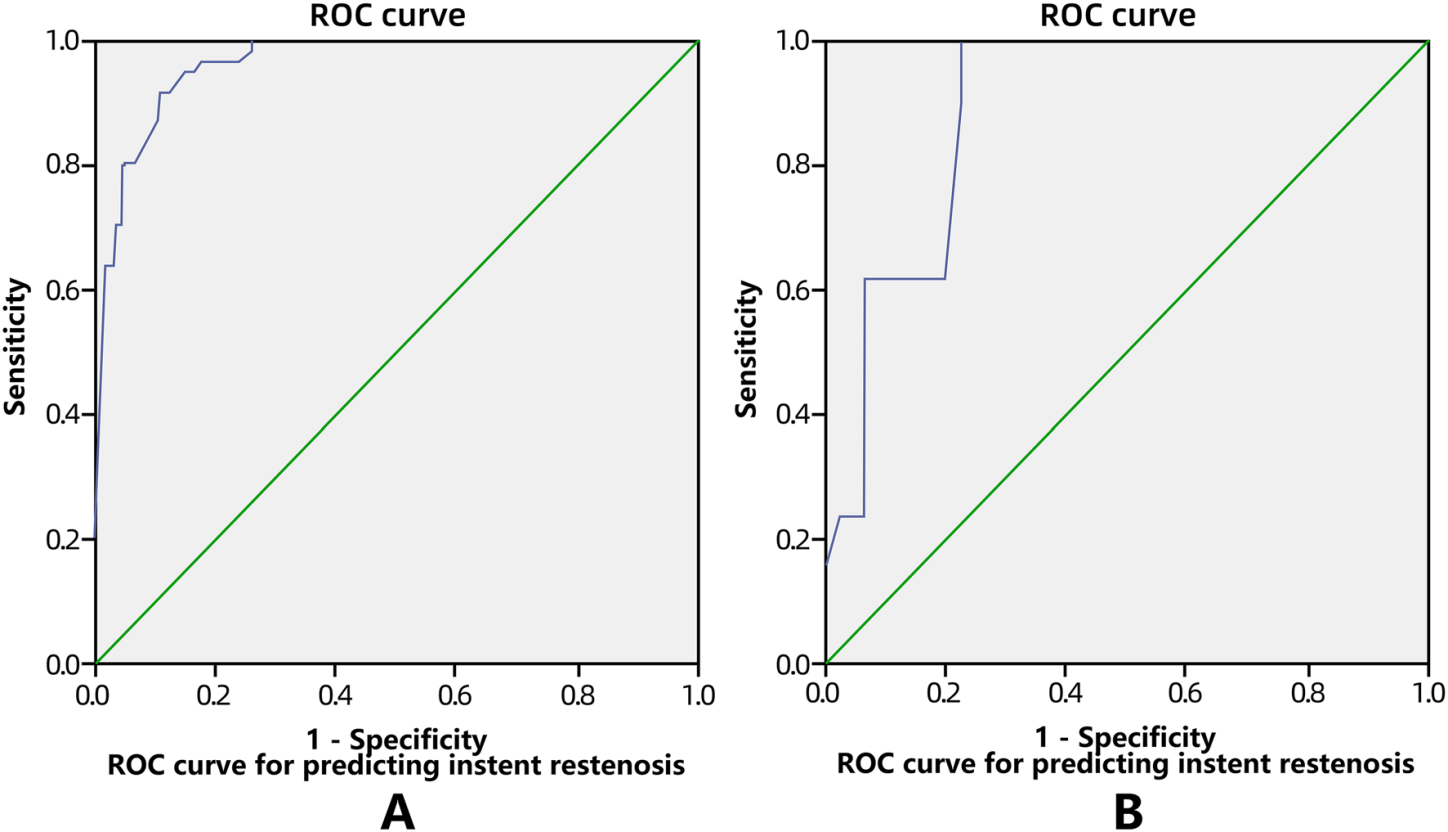
Receiver operating characteristic (ROC) curve for predicting instent restenosis based on imaging and clinical features in the modelling (**A**) and validation (**B**) group.

Based on multivariate analysis in the modeling group, the in-stent restenosis probability prediction model was established as follows: *P* = 1/[1 + e^−( - 6.070–1.391 location+2.745 calcification+4.117 balloon inflation pressure+2.195 intraprocedural dissection+1.163 residual stenosis+1.174 flow TC grade)^]. In this equation, e is the natural constant. In the modeling group with 359 lesions in the first angiographic follow-up, the ROC curve was established using the equation to calculate the probability of in-stent restenosis in each case for comparison with the angiographic follow-up outcome (Fig. 5A). The AUC in the receiver operating characteristic analysis was 0.963 (95% CI: 0.945–0.982), and the sensitivity and specificity of this equation were 78.69% and 95.64%, respectively, when the prediction cutoff value was 0.50. The logistic regression model was validated in the validation group (n = 89), and in-stent restenosis was predicted with this equation for all patients with follow-up. The AUC was calculated as 0.902 (95% CI = 0.836 − 0.969) (Fig. 5B), and the Youden index was 0.7890. When the same cutoff value of 0.50 was adopted, the sensitivity was 76.92%, the specificity was 80.26%, and the total coincidence rate of the model was 79.78% (71/89) (Table 4), indicating the good clinical application value of this model.

**Table 4:** Prediction of instent restenosis based on imaging and clinical features of lesions in the validation group (n = 89).

Restenosis predicted by restenosis equation	Follow-up angiographic outcome		Total
	Restenosis	Non-restenosis	
No	3	61	64 (71.9%)
Yes	10	15	25 (28.1%)
Total	13 (14.6%)	76 (85.4%)	89

## Discussion

In this study, it was found that lesion location, calcification degree, balloon expansion pressure, residual stenosis, intraprocedural arterial dissection, and cerebral blood flow TICI grade were the factors significantly affecting in-stent restenosis after Enterprise stent angioplasty for intracranial atherosclerotic stenoses. The in-stent restenosis prediction model established using these factors was validated to be able to effectively predict in-stent restenosis with good sensitivity and specificity.

Although the Enterprise stent has improved properties with regard to treating intracranial vascular diseases^20^, in-stent stenosis still occurs within the stent. Feng et al^4^ found > 50% in-stent restenosis in 6.81% (three) of patients 22 months after deployment of the Enterprise stent even though the initial symptomatic intracranial arterial stenosis was decreased from 79.3 ± 8.1% to 14.9 ± 12.3% immediately following stenting. In the study with 189 patients harboring 209 intracranial stenoses treated with the Enterprise stent investigated by Vajda et al^13^, in-stent restenosis was observed in 43 (24.7%) cases out of 174 stenoses with angiographic follow-up at a mean time of 10 months even though the poststenting stenosis rate was reduced to 25.1 ± 1% from 65.4 ± 1% before stenting. Salik et al. also detected in-stent restenosis in two (3.3%) of 60 patients with available angiographic follow-up 22 months after deployment of the Enterprise stent for symptomatic high-grade intracranial stenosis, which was decreased from presenting to poststenting (92 ± 6% vs. 12 ± 10%)^12^. Our study identified some significant factors affecting the in-stent restenosis of the Enterprise stent in treating intracranial atherosclerotic diseases, and the logistic model constructed with these significant risk factors can be used to effectively predict in-stent restenosis, which is a major drawback of intracranial stent angioplasty for atherosclerotic diseases.

Hypertension and diabetes mellitus can chronically affect the atherosclerotic process but do not acutely affect in-stent stenosis. During stent-assisted coiling of wide-necked intracranial aneurysms, a certain rate (10.2%-32.12%) of in-stent stenosis can also happen^4,23,24^, with some studies displaying in-stent restenosis as a significant dynamic and spontaneously resolvable process^23,24^. These studies may indicate that in-stent stenosis or restenosis in stents assisting coiling embolization of cerebral aneurysms is not the progression of atherosclerosis but rather the result of intimal hyperplasia. In stent angioplasty for intracranial atherosclerotic stenosis, balloon inflation and stent deployment can tear plaques and damage the intima of the stented artery, leading to an inflammatory reaction of endothelial and smooth muscle cells^23,26^. The intimal reaction to the stent as a foreign body can also cause thrombosis within the stent, which, together with the inflammatory reaction, can bring about in-stent restenosis. Balloon inflation will damage not only the intimal and medial membrane but also the adventitial nourishing blood vessels, and injury of the adventitial nourishing vessels plays an important role in in-stent restenosis. Therefore, balloon inflation pressure should not be too high, and the diameter of the balloon should not be too large to avoid damage to the artery wall. The diameter of a balloon selected for inflating the stenotic segment of the artery should be 80% of the normal artery diameter near the stenotic segment, and optimal expansion of the stenotic artery should not be pursued to prevent damage to the adventitial nourishing blood vessels^13^.

The lesion nature rather than the type was a significant risk factor for in-stent restenosis in our study. Calcification in the lesion can significantly affect in-stent restenosis. For patients with apparent plaque calcification, the Enterprise stent may not be suitable for stent angioplasty because of its low radial force. In atherosclerosis progression, plaque calcification develops via inflammation-dependent mechanisms involved in the progression and regression of atherosclerosis^27^. Intracranial atherosclerotic calcification is not isolated and frequently coexists with arterial angulation or tortuosity^28^, which results in a poor reaction to balloon angioplasty. In plaque calcification, the compliance of the arteries is very poor and may need a greater balloon inflation pressure, which may cause a greater rate of arterial dissection and rupture compared with noncalcified plaques. For severe calcification, the artery may not be expanded sufficiently, and in such insufficiently expanded arteries, the Enterprise stent will not be easily or totally expanded, leading to poor adherence to the wall or irregular deformation of the stent^4^.

Intraprocedural arterial dissection is one risk factor for in-stent restenosis. Based on our experience with carotid stenting, the use of a small balloon for predilation and suboptimal balloon angioplasty before stenting are better for reducing arterial dissection. Follow-up studies revealed that intraprocedural arterial dissection could significantly increase the in-stent restenosis rate. Therefore, the balloon used for dilatation should be selected with the balloon diameter being 80% that of the adjacent normal artery to prevent dissecting the atherosclerotic plaque. Residual stenosis may also increase the in-stent restenosis rate, and a higher rate of residual stenosis was permitted during the procedure to increase the safety of intracranial stent angioplasty. The cerebral blood flow TICI grade is used to evaluate the recanalization degree of occluded arteries with four grades: zero, minimal, partial, and complete recanalization, which significantly affects residual stenosis after stent deployment and subsequently in-stent restenosis at follow-up.

Regarding lesion location in affecting restenosis, the in-stent restenosis rate was significantly higher in the vertebral and internal carotid arteries than in the basilar and middle cerebral arteries (78.4% vs 21.6%, *P* = 0). The Enterprise stent is a closed-cell intracranial stent with low adherent capability in tortuous arteries^29^, which may lead to greater intimal hyperplasia and consequently greater in-stent restenosis in the vertebral and internal carotid arteries. Ebrahimi et al.^29^ found that the Enterprise stent with closed cells has less flexibility to conform to a curved or irregular vessel because the unsegmented closed-cell design does not permit the stent to extend at the outer curvature or to shorten at the inner curvature, resulting in maladaptation to the vascular curvature and flattening or even kinking of the stent in acute curves. This maladaptation will cause intimal hyperplasia in addition to long-term material fatigue damage.

Some limitations existed in this study: we included only participants of Chinese ethnicity in our population, and this was a single-center, retrospective study. Future studies will have to resolve these issues for better outcomes.

In conclusion, in-stent restenosis after Enterprise stenting for atherosclerotic arterial stenosis is affected by stenosis location, calcification, balloon inflation pressure, intraprocedural arterial dissection, residual stenosis and cerebral flow grade, and establishment of a logistic model with these factors can effectively predict in-stent restenosis.

## Materials and methods

This study was approved by the ethics committee of Henan Provincial People’s Hospital. Informed consent was obtained from all patients to participate in the study. All methods were performed in accordance with the relevant guidelines and regulations. Between January 2017 and March 2019, consecutive patients with angiography-confirmed intracranial atherosclerotic diseases (≥ 50% stenosis) who were treated with the Enterprise stent were enrolled. The exclusion criteria were patients with identified or suspected vasculitis or vascular dissection. A total of 400 patients were enrolled in the modeling group to establish a prediction model for in-stent restenosis, and 89 patients with matched baseline data were enrolled for validation of the prediction model (Table 1). The endovascular procedure (Figs. 1, 2, 3, and 4) was performed under general anesthesia with oral administration of clopidogrel (75 mg/d) and aspirin (300 mg/d) at least three days before stenting. All procedures were performed by surgeons with over five years of experience with endovascular techniques. After a 6F guiding catheter was introduced into the vessel, and angiographic evaluation was performed for the atherosclerotic lesion, including location, length and vascular diameter. Then, a Transcend microwire (Transcend, 0.014, 205 cm, Stryker) was navigated together with a microcatheter through the lesion into the distal vessel. An exchange microwire was sent across the lesion, and a Gateway balloon catheter was introduced to the stenosis or occlusion location and gradually inflated to full expansion for 30 s using nominal pressure. The Gateway balloon was selected with the balloon diameter reaching approximately 80% of the diameter of the normal vessel adjacent to the lesion to be treated, and underdilation was recommended to avoid arterial dissection, vessel rupture, and plaque displacement. After inflation, the Gateway balloon catheter was withdrawn, and an Enterprise stent with a diameter of 4.5 mm (common specification of the stent) was sent via a microcatheter to the lesion and deployed to cover the target lesion. The length of the Enterprise stent was selected so that the stent covered the whole lesion and extended approximately 1–2 mm beyond each end of the lesion. After stent deployment, angiographic evaluation was performed for residual stenosis and blood flow through the stent. Technical success was defined as residual stenosis < 50%. If the residual stenosis was > 50%, poststenting dilatation was performed with a balloon catheter. For recanalization of occluded intracranial arteries, a microguidewire was first used to explore the occluded segment, and after the wire was sent through the occluded segment into distal branches, a microcatheter was navigated along the microguidewire through the occluded segment into the distal arterial branches. Angiography was performed through the microcatheter to show whether the microcatheter was in the distal arterial branch. Later, an exchange microwire was sent across the lesion for introduction of a balloon catheter for angioplasty and stenting. After the endovascular procedure, computed tomography was conducted to rule out intracranial hemorrhage or thrombosis. Antiplatelet treatment was conducted with aspirin 100 mg/d and clopidogrel 75 mg/d. Six to 12 months after the procedure, clopidogrel was stopped while aspirin was continued^14,17^.

After stent angioplasty, the following data were recorded and analyzed: the stenosis location, length and degree; normal vascular diameter distal and proximal to the lesion; lesion type; balloon size; inflation pressure and duration; stent type; residual stenosis; perioperative complications; morbidity; and mortality within 30 days. All patients were followed up within 30 days after stenting for possible stroke, death, and transient ischemic attack (TIA). At 1, 3 and 6 months after discharge, clinical evaluation of the patients’ neurological system was performed by means of telephone contact and outpatient services. Angiographic follow-up was performed once between 3–12 months after stenting, and telephone follow-up was performed once yearly afterwards. Head CT angiography (CTA), magnetic resonance imaging (MRI), or digital subtraction angiography (DSA) were performed for those with suspected stroke once a year. For stable patients, the follow-up interval might be longer than two years. Recanalization of the lesion was evaluated before and after the procedure with the thrombolysis in cerebral infarction grade (TICI) grading system^18^, which classifies the recanalization of the occluded artery into 0—no recanalization, 1—minimal, 2—partial, and 3—complete recanalization. The definition of intracranial arterial stenosis provided by Samuels et al^19^ was adopted, and the stenosis was measured using the equation percent stenosis = [(1 − (Dstenosis/Dnormal))] × 100, where Dstenosis indicates the arterial diameter at the location of the most severe stenosis and Dnormal indicates the diameter of the proximal normal artery. According to the study by Levy et al^8^, in-stent restenosis refers to a lesion demonstrating stenosis greater than 50% adjacent to or within the stent and absolute luminal loss greater than 20% on follow-up imaging.

The following variables were analyzed. Lesion calcification was evaluated in terms of location and degree, and the section of the narrowest lesion on CTA was used to evaluate the arterial calcification degree. Calcification location: 0, basilar artery or middle cerebral artery; 1, internal carotid artery siphon segment or vertebral artery V4 segment. Calcification degree: 0, calcification arc ≤ 180°; 1, calcification arc > 180°. Lesion length: 0, ≤ 15 mm; 1, > 15 mm. Balloon inflation pressure: 0, ≤ 4 ATM; 1, > 4 ATM. Intraprocedural arterial dissection following balloon expansion: 0, no dissection; 1, with dissection. Residual stenosis: 0, with residual stenosis ≤ 30%; 1, residual stenosis > 30%. Cerebral blood flow grade: 0, TICI ≤ 2b; 1, TICI > 2b.

## Statistical analysis

The statistical analysis was performed with SPSS 19.0 software (IBM, Chicago, IL, USA). All continuous data are expressed as the mean ± standard deviation and were tested with paired t test and single-factor analysis. Categorical data are expressed as frequencies and were tested with the chi-square test or Fisher’s exact test. The prediction model was identified with multivariate binary logistic regression analysis. A forward-conditional method was used to identify variables significant (*P* < 0.05) for predicting intracranial atherosclerotic lesions. Based on the selected imaging and clinical variables, a simple diagnosis equation was established: P (in-stent restenosis probability) = 1/[1 + e^−z^] for calculation of the in-stent restenosis probability compared with the follow-up data of CTA and digital subtraction angiography. The specificity and sensitivity of the prediction model were evaluated, and the receiver operating characteristic (ROC) curve and the area under the curve (AUC) were calculated in both the modeling and validation groups. A *P* value less than 0.05 was considered statistically significant.
